# Romidepsin Used as Monotherapy in Sequence with Allogeneic Stem Cell Transplant in a Patient with Peripheral T-Cell Lymphoma

**DOI:** 10.1155/2014/404078

**Published:** 2014-07-07

**Authors:** Nicholas Finn, Jean-Francois Larouche

**Affiliations:** ^1^Dr. Léon-Richard Oncology Centre, 37 Providence Street, Moncton, NB, Canada E1C 8X3; ^2^CHU de Québec, Hôpital Enfant-Jésus, 1401 18e Rue, Quebec, QC, Canada G1J 1Z4

## Abstract

Despite advances in the field, a clear treatment algorithm for most peripheral T-cell lymphoma (PTCL) subtypes remains to be defined. Generating reliable randomized data for this type of pathology remains a challenge because of the relative rarity of the disease and the heterogeneity of subtypes. Newer agents, such as the class-I selective histone deacetylase inhibitor romidepsin, have demonstrated efficacy and manageable toxicity in the relapsed and refractory setting. Whether novel agents should be used in conjunction with more conventional cytotoxic therapies or in sequence with a transplant strategy is unknown at this time. Here we report the successful use of romidepsin monotherapy as a bridge to allogeneic stem cell transplantation in a patient who had previously relapsed after several lines of conventional cytotoxic therapy for PTCL. Romidepsin provided the patient with sufficient disease control to proceed to transplantation while remaining in complete remission.

## 1. Introduction

Coiffier et al. reported a 25% overall response rate, including 15% complete response/unconfirmed complete response, in 130 patients receiving romidepsin for relapsed/refractory PTCL in a phase 2 clinical trial [1]. Other investigators have also reported similar response rates (overall response rate, 38%; complete response/unconfirmed complete response, 18%) in a smaller phase 2 trial involving a heavily pretreated population, 38% of whom had already undergone stem cell transplantation [2]. Responses were durable with this agent, as demonstrated by a median duration of response of 28+ months (median follow-up, 22.3 months) in the pivotal trial [3]. On the basis of these data, romidepsin was approved by the Food and Drug Administration in the United States for the treatment of PTCL in patients who have received at least one prior therapy. Because the role of allogeneic stem cell transplantation (alloSCT) in PTCL is still controversial [4–6], little has been published concerning the use of romidepsin as a bridge to this more definitive type of therapy.

## 2. Case Presentation

We report the case of a 44-year-old woman who presented in April 2011 with a 2-month history of significant cervical lymph nodes. Written informed consent was obtained from the patient for publication of this case report. She had associated B-symptoms in the form of weight loss and night sweats. Examination showed that the patient had an Eastern Cooperative Oncology Group status of 1 and no other palpable lymph nodes or spleen enlargement. Results of a surgical lymph node biopsy were compatible with PTCL not otherwise specified; the T-cell infiltrate was positive for CD45, CD3, CD5, CD4, and CD30 and negative for CD20, CD15, and ALK-1. The Ki-67 index was determined to be 30%. Computed tomography (CT) imaging and bone marrow examination indicated stage IIIB disease. Lactate dehydrogenase was 4 times the upper limit of normal, and the complete blood count (CBC) results were as follows: white blood cell count, 16.1 × 10^9^/L (absolute neutrophil count, 8.7 × 10^9^/L); hemoglobin, 110 g/L; and platelets, 43 × 10^9^/L.

The patient was given 8 cycles of CHOP-21 therapy (cyclophosphamide, doxorubicin, vincristine, and prednisone given over 21-day cycles). At the end of treatment, positron emission tomography–CT scanning showed remaining doubtful fluorodeoxyglucose uptake above baseline in 2 cm bilateral lymph nodes in the inguinal regions, and the CBC was normal. Within 3 months of the end of CHOP-21 therapy, the patient experienced disease progression complete with pancytopenia from bone marrow invasion, circulating atypical lymphocytes, hypercalcemia, low-grade renal insufficiency, and B-symptoms. We thought that she might be a good candidate for autologous stem cell transplantation if she proved to be chemotherapy-sensitive. She was given 2 cycles of DHAP (dexamethasone, cytarabine, and cisplatin) that were complicated by an episode of moderate-grade renal insufficiency as well as palpable purpura. Renal biopsies showed a nonspecific lymphoid infiltrate, and skin biopsies showed the presence of nonspecific intravascular thrombi. The patient's kidney failure resolved with corticosteroid treatment, and she was able to complete a third and final cycle of DHAP (with cisplatin substituted by carboplatin) without further kidney compromise. Repeated CT imagery demonstrated a complete response at this point.

Within 3 weeks of the end of second-line therapy, the patient developed a febrile state. Early relapse of disease was determined by CT imagery, which was evidence of progressive pancytopenia with bone marrow invasion and splenomegaly. After 2 nonsustained responses to chemotherapy, nonconventional agents were considered as treatment options; we attempted to subcutaneously administer alemtuzumab at a test dose of 3 mg after prophylactic medication; however, 3 attempts were aborted because of severe allergic reactions.

The patient maintained an adequate performance status throughout the disease course and was discharged to her home for 1 month while attempts were made to obtain romidepsin. Romidepsin, not yet approved by Health Canada, was obtained as monotherapy through a special access program. The first dose of the medication was given on August 23, 2012, following prophylactic antiemetic treatment. Because of preexisting thrombocytopenia, romidepsin was initiated at a reduced dose of 10 mg/m^2^ given on days 1, 8, and 15 of a 28-day cycle. The patient's baseline electrocardiogram was normal, including the QTc. The baseline CBC showed the following: white blood cell count, 3.6 × 10^9^/L; absolute neutrophil count, 1.4 × 10^9^/L; hemoglobin, 67 g/L with red blood cell transfusion dependence; and platelets, 62 × 10^9^/L. Dose intensity was maintained despite some aggravation of the thrombocytopenia midcycle (platelet nadir, 27 × 10^9^/L on day 8 of cycle 1), which recovered prior to the next cycle.

On romidepsin, the patient progressively regained her energy level. She became transfusion-independent, and her spleen became nonpalpable. At cycle 3, the dose of romidepsin was increased to the standard 14 mg/m^2^ because day 1 platelets had improved to 101 × 10^9^/L. The patient no longer had circulating atypical lymphocytes, and her neutropenia was resolving. She required occasional intravenous magnesium supplements on the days of romidepsin therapy to keep electrolytes within the normal range, but she remained in outpatient care. After 4 cycles of romidepsin therapy, restaging with CT imagery and bone marrow sampling demonstrated no evidence of disease.

Because of prior short-duration responses with conventional cytotoxic agents, a transplant team evaluation was requested. The patient was known to have a fully matching sibling donor; therefore, it was thought that her best curative option would be familial alloSCT. A reduced-intensity conditioning regimen was selected due to comorbidities, including diabetes and renal failure with creatinine clearance of 70 mL/min. In preparation for transplant, romidepsin was continued for a total of 8 cycles; the last dose was given 10 days prior to her induction regimen of intravenous fludarabine 30 mg/m^2^× 5 days and intravenous cyclophosphamide 300 mg/m^2^× 5 days. The patient was also given cyclosporin and mycophenolate mofetil for graft-versus-host disease (GVHD) prophylaxis. She proceeded to transplant in late March of 2013 and has been monitored since. Engraftment occurred rapidly, with complete conversion to donor chimerism at day 29. She experienced no acute GVHD and limited chronic GVHD (of the mouth). There were no posttransplant complications, including infection or venooclusive disease. As of 14 months after transplant, the patient remains in remission with no evidence of relapse. Although graft-versus-lymphoma (GVL) effect is controversial, we believe it may have been involved in her sustained remission. Romidepsin was a successful bridge to alloSCT, but there was no evidence that it played a role in GVHD or a potential GVL effect in this patient.

## 3. Discussion/Conclusion

Cases of durable complete remissions with single-agent romidepsin have been well documented in phase 2 clinical trials of patients with relapsed/refractory PTCL [1–3]. Some patients from these trials have gone on to receive SCT; however, the outcomes of these patients have not been reported. To our knowledge, this was the first documented report of romidepsin use in sequence with a nonmyeloablative alloSCT strategy. Although alloSCT is supported by some groups [4, 6], its exact role and timing in PTCL remain to be defined and are actively being investigated by others [5, 6]. We believe that, on a case-by-case basis, newer therapies such as this may be considered in the treatment algorithm of relapsed/refractory PTCL for patients seeking eventual curative options such as alloSCT.
